# The Role of Caveolin 1 in HIV Infection and Pathogenesis

**DOI:** 10.3390/v9060129

**Published:** 2017-05-26

**Authors:** Ayalew Mergia

**Affiliations:** Department of Infectious Diseases and Pathology, University of Florida, Gainesville, FL 32611, USA; mergiaa@ufl.edu; Tel.: +1-(352)-294-4139; Fax: +1-(352)-392-9704

**Keywords:** HIV, caveolin 1, caveolae, cholesterol, macrophages, persistent infection, endothelial cell dysfunction

## Abstract

Caveolin 1 (Cav-1) is a major component of the caveolae structure and is expressed in a variety of cell types including macrophages, which are susceptible to human immunodeficiency virus (HIV) infection. Caveolae structures are present in abundance in mechanically stressed cells such as endothelial cells and adipocytes. HIV infection induces dysfunction of these cells and promotes pathogenesis. Cav-1 and the caveolae structure are believed to be involved in multiple cellular processes that include signal transduction, lipid regulation, endocytosis, transcytosis, and mechanoprotection. Such a broad biological role of Cav-1/caveolae is bound to have functional cross relationships with several molecular pathways including HIV replication and viral-induced pathogenesis. The current review covers the relationship of Cav-1 and HIV in respect to viral replication, persistence, and the potential role in pathogenesis.

## 1. Introduction

Human immunodeficiency virus (HIV) has an extraordinary survival advantage in the host, confounding existing therapies. Anti-retroviral therapy (ART) suppresses HIV replication to undetectable levels and has been effective in prolonging the lives of HIV infected individuals. The suppression of viral replication by ART in many patients raised hopes for virus eradication. However, ART is not capable of eradicating HIV from infected individuals mainly due to HIV’s persistence in small reservoirs of latently infected resting CD4+ cells [1,2,3,4,5,6]. Furthermore, the rise of drug-resistant viruses in some patients under ART treatment remains a concern [7,8,9,10,11,12]. In addition, ultrasensitive assays show that many patients under ART still have low-level viremia [13,14,15,16]. A complete understanding of where HIV persists, and how latency is established and maintained in patients on ART is critical in developing strategies to cure HIV infection. Although more work is still needed, there are significant developments in our understanding of a persistent HIV infection in patients under drug therapy.

Latently infected resting memory CD4+ cells are present in the blood and lymphoid tissues of patients under ART [17,18]. In blood, central and transitional memory cells are the predominant population of latently infected cells [19]. Albeit at a lower frequency, persistent HIV DNA is also found in naive CD4+ T cell populations of patients on ART [19,20]. Residual viral reservoirs in the gastrointestinal (GI) and genitourinary tract as well as lymphoid tissues in patients under drug treatment can be an additional obstacle for curing HIV [20,21,22,23,24,25,26]. Recent evidence also shows HIV infection in multi-potent hematopoietic progenitor cells in the bone marrow where some of the cells are latently infected [27,28]. Furthermore, it has been confirmed that monocytes, dendritic cells, and macrophages can be infected latently or with a low-level replication and can contribute to viral persistence [29,30,31,32,33,34].

HIV persistence in monocytes/macrophages helps the virus disseminate into the central nervous system. Initial brain infection is thought to occur through an infected monocyte-macrophage traversing the blood-brain barrier [35]. Infected monocytes that enter the brain can then repopulate the resident macrophages with subsequent infection of the existing resident macrophages, microglial cells [36,37,38], astrocytes [38,39,40], and possibly pericytes [41]. Productive HIV replication within the brain mainly occurs in microglial cells and perivascular macrophages whereas the virus does not productively infect neurons [37]. Many drugs are restricted from crossing the blood brain and blood cerebrospinal fluid barrier and are therefore suboptimal in penetrating the central nervous system [42,43]. Consequently, the virus can persist in these cells serving as a reservoir and can escape the immune system and ART. Furthermore, infected macrophages are present in lung and duodenal tissues of patients receiving ART [44,45,46]. Macrophages are therefore important HIV targets that can serve as a sanctuary for viral persistence. However, less attention has been given to macrophage persistent infection compared to studies of latent and persistent infection in T cells. This could be due to the difficulties of accessing macrophages in human tissues for thorough investigations.

The induction of a progressive depletion of T cells leading to immune dysfunction and the formation of a viral reservoir are a hallmark of HIV infection [47,48]. The cytopathic effects that lead to T-cell depletion are induced by several mechanisms. Infected macrophages, on the other hand, are relatively resistant to the cytopathic effect and consequently play an essential role in viral dissemination to host tissues and organs [49,50,51,52,53,54]. Current evidence indicates that the sustained persistent infection of macrophages, contrary to the productive infection of CD4+ T cells, is due to differences with HIV protein trafficking and virus release between the two cell types [55,56,57]. In macrophages, HIV assembles and matured viral particles accumulate in intracellular compartments [56,58,59,60,61,62,63]. Other studies have described the plasma membrane to be the primary site of virus assembly and budding in macrophages [64,65,66]. Emerging studies brings these two different observations together and reveal that HIV assembles and accumulates in intracellular compartments and is connected to the extracellular space [67,68]. Intracellular virus maturation in macrophages protects HIV from immune responses and helps the virus to persist in patients under ART. Furthermore, in this viral reservoir, HIV infection appears to not be associated with apoptosis but with a chronic productive infection [69,70]. In support of this observation, evidence has been established for chronically infected circulating monocytes in which there is a steady stable expression of antiapoptotic genes [71]. The half-life of infected macrophages is, therefore, substantially longer than that of T cells [53,54]. As a result, these cells continue to accumulate replication-competent HIV for prolonged periods of time, even in patients receiving ART [52]. We still do not have a complete picture as to the mechanism by which these persistently infected cells can promote an antiapoptotic environment while T cells cannot. Furthermore, it is not clear whether a lack of apoptosis is related to persistent infection in macrophages.

The relationship between HIV and host factors determines the modulation of cellular function and replication of the virus within an infected individual. Interaction of these viral and cellular proteins is evident in all steps of virus replication and their associations may be an important factor in the modification of host cell functions during a chronic viral infection [49,52,72,73,74]. HIV has also managed strategies that counter host innate viral restriction factors and consequently establish persistent infection [75,76,77,78,79]. There is a growing list of restriction factors that suppresses virus replication which include apolipoprotein B messenger RNA (mRNA) editing enzyme catalytic polypeptide-like (APOBEC) 3F and G, tripartite motif protein 5 alpha (TRIM5α) and TRIMcyp, sterile alpha motif histidine/aspartic 1 (SamHD1), and tetherin [75,76,77,78,79]. These factors restrict HIV at different stages of replication. APOBEC gets packaged into viral particles and inhibits viral copy DNA (cDNA) synthesis by affecting the processivity of reverse transcription and by causing the inactivation of G-to-A hypermutations in the proviral genome [80,81]. TRIM5α targets incoming HIV capsids and blocks viral replication by preventing viral cDNA synthesis [82]. Thetherin is a type II transmembrane protein that restricts the propagation of many enveloped viruses including HIV by tethering progeny budding virions onto the plasma membrane [83,84]. This consequently prevents virus release. SamHD1 suppresses reverse transcription through the depletion of nucleoside pools required for effective cDNA synthesis [85,86]. It may also repress reverse transcription by degrading viral RNA. HIV and simian immune deficiency virus (SIV) have managed to counter these restriction factors by accessory proteins. The HIV viral infectivity factor (VIF) protein, which is essential for effective propagation of the virus in vivo, prevents the encapsidation of APOBEC into virions [81]. The antagonist to tetherin is the viral protein U (Vpu) for the HIV-1 strains and viral negative factor (Nef) for other primate lentiviruses [78,83,84]. Viral protein X or R (Vpx or Vpr) counter the antiviral activity of SAMHD1 for HIV-2 and SIV [85,86]. Additional cellular antiviral factors with relevance beyond HIV include interferon-induced transmembrane (IFITM) proteins, cholesterol 25-hydroxylase (CH25H), KRAB-associated protein-1/TRIM28 (KAP1/TRIM28), 90K, putative helicase moloney leukemia 10 homolog (MOV10), interferon-induced GTP-binding protein (MxB), Schlafen family member 11 (SLFN11), and zinc-finger antiviral protein (ZAP) [76]. HIV can also counter these antiviral activities and establish infection. Therefore, the persistent infection and survival of macrophages is potentially regulated by the contest between viral and cellular factors at different steps in virus replication, implying that there may be several mechanisms for HIV longevity in macrophages. Gene expression profiles that are unique to macrophages [71,87,88] when compared to those of activated CD4+ T cells should help determine the mechanism of persistent infection in macrophages. One molecule with multiple functions that is expressed in macrophages while being absent in T cells is caveolin 1 (Cav-1). This review will cover the functional importance of Cav-1 in HIV replication and its potential contribution to HIV persistence and pathogenesis.

## 2. Caveolin 1 and Its Function

Caveolin 1 (Cav-1), a 21–24-kDa scaffolding protein, is an important structural component of the caveolae organelle [89]. Caveolae are flask-shaped small invaginations of 50–100 nm diameter in the plasma membrane that are highly enriched in cholesterol, phospholipids, and sphingolipids, and are abundant in various cell types [90,91,92,93,94]. Cav-1 is highly expressed in terminally differentiated or quiescent cells including muscle cells, adipocytes, endothelial cells, monocytes, macrophages, dendritic cells, microglia, and astrocytes, while human T cells appear to lack the Cav-1 protein [90,91,92,93,94,95,96,97,98,99,100,101]. Along with Cav-1, the caveolae structure is composed of Cav-2 and Cav-3 as well as four additional proteins known as cavin 1–4 [102,103,104]. Cav-1 and Cav-2 are found in abundance in most cells of the cardiovascular system, whereas Cav-3 is limited to skeletal muscle and some smooth muscle cells [103,105,106]. For caveolae structural integrity, Cav-1 or Cav-3 is important, while Cav-2 is dispensable [99,107,108,109,110]. The role of Cav-2 is less well defined. Cavins are important as regulators of caveolin and are required for the assembly and function of caveolae [104]. Recent electron cryomicroscopy studies revealed a regular polyhedron architecture of caveolae with cavins [111]. The caveolar architecture is connected with unstructured cavin filaments by coiled-coil domains into a polygonal net-like complex. This complex is believed to provide scaffolding for compartmented cellular processes and participates in multiple cellular functions, including endocytosis, transcytosis, membrane homeostasis, inflammation, and signal transduction [94,104,111,112,113,114,115,116,117,118,119,120,121]. 

The mechanisms of the multiple functions described for caveolae are not fully understood and remain controversial. These are further complicated by the findings and emerging evidence that caveolae serve as mechanosensing organelles and may play a role in the protection of cells from mechanical stress by stretching or internalizing the caveolae structure [122,123,124,125]. Caveolae are present in abundance in mechanically stressed cells such as muscle cells, fibroblasts, endothelial cells, and adipocytes compared to other cell types, strengthening their role in the protection of cells from mechanical stress [124,126]. Therefore, the many different functions described previously have to be reexamined in concert with the model of mechanosensing to establish a complete understanding of the role of caveolae in cell physiology. 

Cav-1 is also present in cells independent of the caveolae structure, suggesting additional cellular functions. Along with being a component of caveolae, Cav-1 is involved in several cellular processes that include cell cycle regulation, cholesterol trafficking and efflux, senescence, signal transduction, angiogenesis, endocytosis, transcytosis, and apoptosis (Table 1) [95,127,128,129,130,131,132]. Multiple lines of evidence indicate that Cav-1 interacts with a variety of signal molecules such as Src tyrosine kinases, glycosylphosphatidylinositol linked receptors, small guanosine triphosphate hydrolyzing enzymes (GTPases, heteromeric G protein, ion channels, endothelial nitric oxide synthase (eNOS), protein kinase A, and mitogen-activated protein kinases [132,133,134]. Cav-1, therefore, acts as a scaffolding molecule and can directly associate and modulate several signal transducing molecules.

## 3. Cav-1 Mediated HIV Inhibition

The multifaceted functions of Cav-1 that include signal transduction, cell division, and cholesterol homeostasis suggest that Cav-1 has functional relationships with virus replications and viral induced pathogenesis. The first evidence that Cav-1 may play a role in HIV replication is the demonstration of the inhibition of HIV particle production from provirus-transfected cells [135]. Further studies showed that the level of group specific antigen (Gag) production was significantly reduced. This observation, and the fact that Cav-1 is expressed in macrophages while being absent in T cells, prompted the idea that Cav-1 can restrict HIV replication and consequently contribute to macrophage HIV persistent infection. Since Cav-1 has multiple functions, the mechanisms of HIV inhibition by Cav-1 can be complex and involve multiple steps in virus replication. The finding that Gag production is reduced in cells expressing Cav-1 suggests that one mechanism of the inhibition of HIV replication by Cav-1 can be the post integration of the proviral DNA. In fact, Wang et al*.* demonstrated that Cav-1 inhibits HIV replication at the transcriptional level in macrophages [136]. Site specific mutational analysis of the HIV long terminal repeat (LTR) cis-acting elements reveals that the nuclear factor kappa-light-chain-enhancer of activated B cells (NF-κB) is important for Cav-1 mediated repression of HIV transcription. The two NF-κB sites in the LTR have to be mutated to abrogate the transcriptional inhibition of Cav-1. Further studies showed that Cav-1 affects the NF-κB pathway by reducing the phosphorylation/activation of the Inhibitor of NF-κB kinase subunit beta (IKKβ), IKK alpha (IKKα), inhibitor of kappa B (IκBα), and p65 as well as subsequent translocation of the NF-κB p65 protein into the nucleus. This consequently decreases the level of p65 binding to target DNA and HIV transcription. The finding that IKKα and IKKβ phosphorylation is reduced implies that the influence of Cav-1 on the NF-κB pathway is upstream of the IKK activation step. Simmons et al. reported similar results showing that Cav-1 inhibits HIV replication by influencing the NF-κB pathway and reducing HIV transcription [137]. Their finding reveals that the mechanism of transcription inhibition is mediated by Cav-1 induced hypoacylation of NF-κB. Further studies by Simmons et al. reveals no reduction in nucleus translocation of NF-κB p65 and binding to target DNA. The discrepancy of these findings with the study by Wang et al., in which they found that Cav-1 reduced nucleus translocation of NF-κB and binding to target DNA, is not clear. However, the discovery by both groups reveals and convincingly establishes that HIV transcription repression by Cav-1 involves the NF-κB pathway. 

The NF-κB pathway is involved in the broad regulation of gene expressions including pro-inflammatory molecules. Cav-1, therefore, can be an important direct and/or indirect regulator of the innate and adaptive immune systems. Immune activation plays a central role in T cell depletion by high turnover of both CD4+ and CD8+ cells, resulting in a shorter half-life of T cells during HIV infection [138,139,140,141]. Increased serum levels of pro-inflammatory cytokines and chemokines are also a consequence of immune activation [142]. The significant gastrointestinal (GI) tract damage due to HIV infection has been linked to immune activation and microbial translocation to the blood circulation with heightened levels of circulating lipopolysaccharide (LPS) [143,144,145,146]. Microbial translocation can influence HIV disease progression partly by the LPS triggering of monocyte activation by way of CD14 and Toll like receptor (TLR) 4-mediated signaling, resulting in a release of soluble CD14 and pro-inflammatory cytokines [145,146]. Further studies suggest a correlation of plasma LPS levels with T-cell activation markers indicating that T-cell activation is an indirect consequence of LPS induced monocyte stimulation, subsequently resulting in the immune activation observed in HIV infected individuals [145,147]. LPS binding to the extracellular domain of TLR4 initiates the recruitment of adaptors triggering signal-transduction cascades, which eventually leads to the activation of transcription factor NF-κB [148,149,150]. The recruitment of adaptors also activates mitogen-activated protein kinases (MAPKs) resulting in the activation of activator protein 1 (AP-1) transcription as well as the activation of interferon response element 3 (IRF-3). These subsequently enhance the transcription of pro-inflammatory molecules, type 1 interferon, and chemokines. Interestingly, LPS is shown to up-regulate Cav-1 expression in macrophages and Cav-1 subsequently blocks the TLR4 pathway by interacting with TLR4 molecules [151,152,153]. The production of pro-inflammatory molecules is tightly regulated by factors that promote the inhibition of enhanced expression, thereby resolving inflammation [149,154,155,156,157]. The finding that Cav-1 suppresses HIV transcription through NF-κB and Cav-1 blocks the TLR4 pathway suggests a role for Cav-1 in regulating inflammatory responses. Although there is an overall induction of inflammatory molecules during HIV infection, Cav-1 may be involved in the regulation of cytokine storms seen after infection.

HIV induced immune activation also leads to the clonal exhaustion of T cells, causing a reduction in memory T cell populations [158,159,160]. Interaction of programed death 1 (PD-1) with its ligand such as PD-L1 or PD-L2 delivers an inhibitory signal to T cells that affects cell proliferation and cytokine production [161]. HIV infection enhances the expressions of PD-1 in T cells and PD-L1 and PD-L2 in macrophages [161,162,163,164]. PD-1 engagement with its ligands has been described to play a role in T cell exhaustion as a result of chronic HIV infection [162,165,166]. The NF-κB signaling pathway is a key participant of the induction of PD-L1 expression in macrophages [167]. Furthermore, LPS up-regulates the expression of PD-L1 through TLR4 mediated NF-κB activation and the four potential NF-κB binding sites on the PD-L1 promoter [167,168]. Since Cav-1 inhibits the NF-κB and TLR4 pathways, it may have an essential role during HIV infection in regulating the PD-1/PD-L1 interactions influencing T cell exhaustion. The role of Cav-1 in regulating the PD-1/PD-L1 pathway, however, could be masked due to continuous antigen stimulations, tipping the balance toward the T-cell exhaustion phenotype.

## 4. HIV Induces Cav-1 Up-Regulation

**Figure 1:**
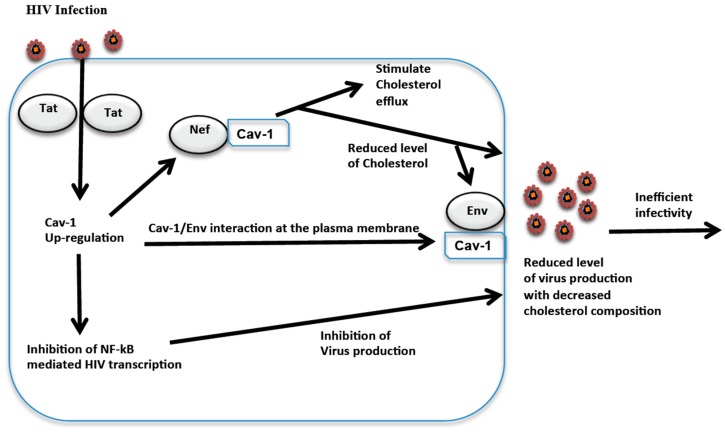
A model for bidirectional interplay between human immunodeficiency virus (HIV) infection and Caveolin 1 (Cav-1) in establishing persistent infection in macrophages by three mechanisms. HIV infection up-regulates the expression of Cav-1 and the enhanced level of Cav-1 subsequently represses virus replication by suppressing the activity of nuclear factor kappa-light-chain-enhancer of activated B cells (NF-κB), countering viral negative factor (Nef) and promoting apolipoprotein A-I (apoA-I) mediated cholesterol efflux, subsequently reducing available cholesterol within the cell, and blocking the fusion steps of virus infectivity. Env: envelope; Tat: transactivator.

The associations of restriction factors with HIV replication may be important in the modification of host cell processes during a chronic viral infection and the establishment of viral persistence. Along with the conventional innate antiviral response, these factors can serve as a first line defense against HIV infection. The counter by the HIV accessory proteins, however, leads to a robust replication of the virus and potentially could contribute to HIV persistent infection in vivo [76,77,78,79]. These restriction factors are constitutively expressed and/or can be induced by interferon during viral replication depending on the cell type [75,76,77]. Low-level constitutive expression of Cav-1 is evident in macrophages, microglial cells, and astrocytes [169,170,171]. It is not clear whether Cav-1 can be induced by interferon during virus infection. However, there are several molecules including LPS that modulate the expression of Cav-1 by different mechanisms. LPS stimulates Cav-1 expression in macrophages and endothelial cells [172]. Similarly, macrophage treatment with oxidized low-density lipoprotein (oxLDL) or simvastatin up-regulates the expression of Cav-1 [173,174]. In addition, the induction or repression of Cav-1 expression has been observed by different cellular factors at the transcriptional level [175,176,177,178]. Interestingly, HIV infection up-regulates the expression of Cav-1 in macrophages [169]. Cav-1 up-regulation by HIV infection and the observation that the overexpression of Cav-1 inhibits virus replication [135,179] suggests that Cav-1 participates in maintaining a low-level persistent infection of macrophages in a feedback loop (Figure 1). Although distinct, such a mechanism of virus repression appears to be similar to the innate immune response where the virus infection stimulates restriction factors and consequently leads to inhibition of virus replication. Similarly, unrelated to HIV, the overexpression of Cav-1 is shown to suppress NF-κB activation in murine macrophages [180]. Furthermore, human cancer cell lines treatment with growth hormone-releasing hormone (GHRH) antagonist MIA-602 show an increase in Cav-1 expression with a consequence of down-modulation of NF-κB [181]. These observations support the finding that the inhibition of HIV replication by suppressing NF-κB activity is due to enhanced Cav-1 expression during HIV infection.

The mechanisms of the stimulation of Cav-1 expression during HIV infection can be complex and indirect influence of viral protein activities on cellular pathways. Lin et al. has shown that HIV infection up-regulates the expression of Cav-1 at the transcriptional level and the HIV transactivator protein (Tat) is responsible for the enhancement of Cav-1 expression [169]. There is limited information as to how Tat up-regulates the expression of Cav-1. Cis-acting elements in the *Cav-1* gene promoter include sequences for the binding transcription factors specific protein 1 (Sp1), transcription factor E2F, and NF-κB, as well as G/C rich box consensus for sterol response elements binding protein 1 (SREBP-1) and p53 [182,183,184,185]. The complex formations and binding of p53, E2F, Sp1, and SREBP1 to the promoter region activates Cav-1 expression [182,183,184]. Thus far, limited analysis of these factors for HIV or Tat mediated Cav-1 up-regulation reveal that reducing the level of Sp1 by targeting with small interfering RNA (siRNA) has no influence [169]. However, p53 activity is essential for Tat mediated induction of Cav-1 expression and correlates with enhanced phosphorylation of p53 Ser residues at amino acid positions 15 and 46. The requirement of p53 activity is not clear and needs further studies to understand the mechanisms of Tat mediated Cav-1 up-regulation. Both LPS and statin promote SREBP activation in macrophages [186,187], and this activation could enhance the SREBP binding to the Cav-1 promoter with the subsequent induction of Cav-1 expression. HIV infection leading to microbial translocations into the blood stream with elevated levels of LPS thus may result in increased Cav-1 expression by inducing SREBP binding to the Cav-1 promoter. Similarly, statins are often used to treat dyslipidemias associated with HIV disease and this may also promote Cav-1 up-regulation by enhanced SREBP interaction with the Cav-1 promoter. 

An important factor that has relevance to Cav-1 up-regulation is oxidative stress which is a central contributing priming cause to many parameters leading to pathogenesis in HIV infected individuals, whether patients are under ART treatment or not. Several studies have shown that oxidative stress enhances the expression of Cav-1 [131]. p38 MAPK, Sp1, and NF-κB, cyclooxygenase 2 (COX2), and phosphatidycholine-specific phospholipase activates *cav-1* gene promoter during oxidative stress resulting in enhanced Cav-1 expression [172,188,189,190,191]. The HIV proteins Tat, Nef, Vpr, and Gp120 have been shown to independently increase reactive oxygen species (ROS) while decreasing antioxidants, establishing HIV-mediated oxidative stress [192]. Interestingly, the inhibition of p38 MAPK can block HIV or Tat alone mediated up-regulation of Cav-1 [169]. Furthermore, the inhibition of p38 MAPK can suppress the phosphorylation of p53 in the presence of Tat and subsequently decreases Cav-1 up-regulation. These findings imply that Tat’s effect on Cav-1 expression is indirect without physical association with the Cav-1 promoter and possibly involves HIV/Tat induced oxidative stress. During HIV infection it is likely that there is a functional cross-link with molecules and pathways that enhance the Cav-1 expression. Zhong et al. described caveolae-associated signaling in the disruption of the cellular tight junctions of the human brain microvascular endothelial cells upon Tat exposure [193]. The disruption in tight junctions correlates with Tat activated RAS and RhoA along with Cav-1 up-regulation, leading a decrease in junction proteins. The silencing of Cav-1 reduced the effect of caveolae associated signaling, implying a role for Cav-1 in the disruption of those cell-to-cell junctions. Human brain endothelial cells co-cultured with HIV infected cells show an induction of phosphorylation of extracellular signal–regulated kinases (ERK) 1/2 and Akt with subsequent activation of Sp1, AP-1, and NF-κB transcription that leads to an increase of the matrix methalloproteinases 9 (MMP-9) promoter and enzyme activity [194,195,196]. Silencing Cav-1 with siRNA treatment attenuates the HIV induced phosphorylation of ERK/Akt and MMP-9 activity. The ERK1/2 and Akt pathways are stimulated in response to oxidative stress, which subsequently enhance the expression of the MMP9 protein [197]. Thus, enhancing Cav-1 expression during HIV infection may involve Tat induced oxidative stress along with other mechanisms of HIV mediated oxidative stress. Such stimulated Cav-1 expression is, therefore, one of the likely host factors that HIV modifies in its effort to establish a persistent infection.

## 5. Cav-1 Mediated Cholesterol Balance and Role in HIV Macrophage Infection

Cholesterol trafficking is a complex event in which the molecular pathways are controlled by several molecules that include Cav-1, SREBP-1, ATP-binding membrane cassette transport protein A1 (ABCA1) and G1 (ABCG1), and scavenger receptor B1 (SR-B1) [198,199,200,201,202,203]. Since Cav-1 is critical in the formation and structural integrity of caveolae, optimal levels of cholesterol are important in caveolae for efficient compartmented cellular processes and downstream signaling [94,204,205,206]. Cholesterol depletion can result in the disruption of efficient communication of the cellular processes [207,208,209,210]. Cav-1 plays an essential role in maintaining cholesterol balance [94,120,204,211,212,213,214]. It mediates the transport of newly synthesized cholesterol from the endoplasmic reticulum (ER) to the plasma membrane [204,205,215,216,217,218,219,220,221] and indirectly influences the transfer to extracellular acceptors such as high-density lipoprotein (HDL) or lipid free apolipoprotein A-I (apoA-I). Cav-1 is, therefore, an important molecule in the maintenance of cellular cholesterol homeostasis. Furthermore, Cav-1 is shown to interact with sterol carrier protein-2 (SCP-2) and this protein is also involved in cholesterol transfer from the ER to the plasma membrane [222,223]. ABCA1 participates in cholesterol efflux to apoA-I through the reorganization of plasma membrane microdomains [224,225,226,227]. 

Cav-1 can influence HIV replication through its role in cholesterol homeostasis. Cholesterol is an important structural component that modulates the fluidity of biological membranes and is critical for the uptake of HIV as well as the budding of virus particles from infected cells [228,229,230,231,232,233,234]. Its depletion significantly reduces HIV particle production. There is also a marked decrease in the infectivity of virions produced from such cells [235]. Therefore, the level of membrane cholesterol composition influences HIV production and virus infectivity. The increase in Cav-1 in macrophages during HIV infection can result in enhanced cholesterol transport to the cell membrane, facilitating increased efflux. This can lead to the depletion of cholesterol, subsequently decreasing virus production. In fact, Lin et al. have demonstrated a reduction in virus production that correlates with enhanced cholesterol efflux in cells overexpressing Cav-1 [236]. Virus particles released from these cells have a decreased cholesterol composition and lower infectivity capacity.

HIV manipulates cholesterol metabolism in order to produce a sufficient supply of cholesterol for efficient virus production [229,235,237,238]. The HIV accessory protein Nef plays an essential role in altering cholesterol metabolism to the benefit of HIV replication [239,240]. Nef impairs ABCA-1 dependent cholesterol efflux by apoA-l [241]. Further studies by Lin et al. reveal that Cav-1 counters Nef and enhances apoA-I mediated cholesterol efflux, resulting in the inhibition of HIV replication [236]. The exact mechanism by which Cav-1 counters impaired cholesterol efflux by Nef is not clear. Nef can down-regulate the expression of ABCA-1 as well as alter its cellular distribution [241]. Such changes in ABCA-1 can impair apoA-I mediated cholesterol efflux. The physical interaction of ABCA-1 and Nef has been established [241,242,243]. However, this interaction does not appear to be important for the down-modulation of ABCA-1. Nef induces degradation of ABCA-1 by proteaosomal activity and/or other undiscovered mechanisms are suggested as a possible explanation for the down-modulation of ABCA-1 [242]. Cav-1 is also shown to co-immunoprecipitate with Nef, implying a potential physical association [236]. It remains to be determined whether this potential association counters the down-regulation of ABCA-1 expression. ABCA-1 is reduced in Cav-1 defective animals, suggesting that Cav-1 positively promotes the expression of ABCA-1 [204]. However, in cells overexpressing Cav-1, the level of ABCA-1 does not change as compared to cells expressing normal levels of Cav-1 [236]. In HIV infected cells, Tat up-regulates Cav-1 while Nef down modulates ABCA-1 expression [169,241]. The mechanism by which Cav-1 restores impaired ABCA-1 dependent cholesterol efflux by apoA-l could therefore be complex and may not have to do with the expression levels of ABCA-1. Nonetheless, it is clear from the studies by Lin et al. [236] that Cav-1 can inhibit HIV replication in macrophages by promoting apoA-I mediated cholesterol efflux and thereby could contribute to a low-level viral replication and persistence in macrophages (Figure 1).

Relevant to the role of Cav-1 in cholesterol mediated HIV inhibition, there is also a significant suppression of envelope (Env) induced membrane hemifusion by Cav-1. Reduction in virion infectivity caused by cholesterol depletion is mainly due to the effect on the fusion step during infection [234,244]. The HIV Env interacts with Cav-1 via the gp41 trans-membrane domain [245,246,247]. This interaction is mediated by the Cav-1 scaffolding domain and WNNMTWMQW in the ectodomain (in the C-terminal heptad repeat region) of HIV transmembrane envelope glycoprotein Gp41 [245,246]. Membrane fraction analysis reveals that the binding of Cav-1 with HIV Env appears to be at the plasma membrane of the caveolae lipid raft [247]. Interaction of these two molecules at the plasma membrane blocks HIV envelope mediated fusion with target cells and reduces virus replication. The ability of Cav-1 to block Env mediated hemifusion and its up-regulation by HIV infection suggests that the Cav-1 interaction with Env, possibly, in concert with enhanced cholesterol efflux, blocks virus production and infectivity (Figure 1). 

## 6. HIV Infection and Influence on Aortic Endothelial Cell Cav-1 Function

Cav-1 plays a key role in several important endothelial cell functions including innate immunity and inflammation, vascular permeability, and leukocyte migrations [153,248,249,250,251,252,253,254]. HIV infection is associated with endothelial dysfunction and increased expression of inflammatory cytokines and adhesion molecules, which results in recruitment and activation of leukocytes and platelets [255,256,257,258]. In addition, HIV infection can lead to pulmonary artery hypertension (PAH) and atherosclerosis [259,260,261,262]. In nonhuman primate models, PAH is observed with the infection of chimeric SIV containing the HIV Nef (SHIV), while the control SIV shows no PAH, suggesting a role for Nef in PAH pathology [263]. Other studies show a significant increase in superoxide release during ex vivo exposure of porcine pulmonary arteries or human pulmonary artery endothelial cells to Nef with concurrent decreases in eNOS and nitric oxide (NO) production [264]. The reduction in NO leads to a decrease in vasorelaxation. In vivo administration of NO can diminish PAH [260,261,262]. Since Cav-1 and caveolae are abundant and play essential roles in endothelial cell function, there has to be link with HIV induced endothelial dysfunction. It is well established that NO is a critical molecule in vascular physiology [265,266,267,268]. Cav-1 is a regulator of eNOS. Depending on cell physiology, Cav-1 inhibits the function of eNOS and negatively regulates the production of NO [269,270,271,272]. Interestingly, mutation in Cav-1 has been linked to generalized lipodystrophy (CGL) and PAH [273,274,275,276]. However, whether there is a direct correlation of HIV or Nef induced PAH with Cav-1 remains to be determined.

**Figure 2:**
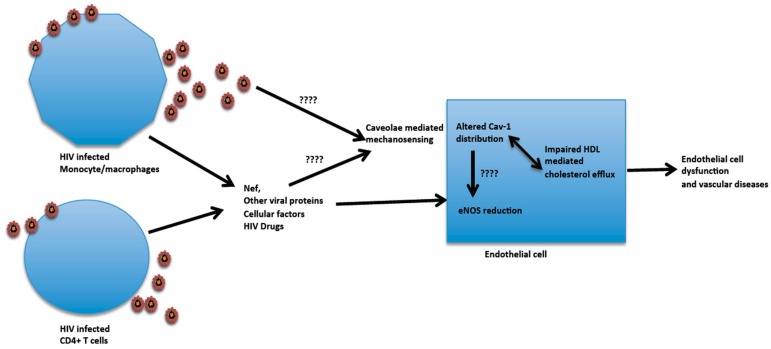
HIV influence on aortic endothelial cell Cav-1 functions and potential link to pathogenesis. Viral proteins released from infected cells in combination with the induction of cellular factors such as cytokines and chemokines in the microenvironment can promote changes in Cav-1 cell distribution that impairs high-density lipoprotein (HDL) mediated cholesterol efflux and endothelial nitric oxide synthase (eNOS) regulation. There is also a reduction in eNOS, which could be related to Cav-1 redistribution. The mechanosensing function of Cav-1 may be an alternative or additional mechanism that leads to Cav-1 redistribution. These HIV induced endothelial cell dysfunctions through Cav-1 can be contributing factors to vascular diseases in HIV infected individuals.

A recent finding show that the co-culture of aortic endothelial cells with HIV infected cells promotes redistribution of endothelial Cav-1 leading to the disruption of HDL mediated cholesterol efflux [277]. Further studies reveal, although with a high dose used, that recombinant Nef exerts similar effects on the redistribution of Cav-1 and impacts the HDL mediated cholesterol efflux. Comparative investigations between wild type and Nef defective HIV reveal no significant differences in the reduction of HDL mediated cholesterol efflux from endothelial cells, suggesting that multiple factors are involved that include Nef to alter cholesterol efflux. The influence of HIV on Cav-1 redistribution and cholesterol efflux can alter cholesterol homeostasis and membrane molecule compositions which can impact caveolae/Cav-1 functions and the caveolae structure. This subsequently could affect the regulation of eNOS by Cav-1 and bioactive NO production, as well as other endothelial cell functions that can lead to HIV induced pathology (Figure 2). Alternatively, Cav-1 redistribution may be related to the mechanostress induced on endothelial cells by HIV and its proteins along with HIV stimulated alterations in cytokine and chemokine productions in the microenvironment. The internalization of caveolae can repair wounded cells and muscle fibers [278]. The Cav-1 redistribution, therefore, could be due to the internalization of caveolae to repair distressed endothelial cells. In support of this notion, pulmonary dysfunction linked to a lack of caveaole is suggested to be a consequence of loss of mechanoprotection [112]. Thus, the cumulative effect of HIV infection with the alteration of the microenvironment could lead to irreversible mechanostress, leading to endothelial cell dysfunction.

In addition to HIV infection, the cardiac risks in acquired immune deficiency syndrome (AIDS) patients have been linked to ART treatments [279,280,281,282,283,284,285,286,287,288], particularly the previously used protease inhibitors [289,290,291]. Ritonavir decreases the expression of Cav-1 and inhibits cholesterol efflux in macrophage-derived foam cells [292]. Nelfinavir also reduces Cav-1 expression in macrophages [293]. Drug induced accumulation of cholesterol within macrophages due to the inhibition of cholesterol efflux could be related to the reduction of Cav-1 expression. This may be one factor attributed to the progression of atherosclerosis in HIV infected individuals under drug therapy. Endothelial cells may suffer from a similar reduction in Cav-1 due to drug therapy, resulting in endothelial dysfunction.

## 7. Conclusions

Cav-1 and the caveolae structure may participate in several steps of HIV infection and pathogenesis. The mechanisms of HIV persistent infection in macrophages are not completely understood. There are three possible mechanisms that Cav-1 could contribute to HIV persistent infection in macrophages. HIV infection up-regulates the expression of Cav-1 and the enhanced level of Cav-1 subsequently represses virus replication by suppressing the activity of NF-κB, promoting cholesterol efflux, and blocking the fusion steps of virus infectivity. Thus, through such bidirectional relationships with HIV, Cav-1 can contribute to a low-level HIV persistence in macrophages. Since Cav-1 is also expressed in microglial and astrocytes along with macrophages, it could be a contributing factor to HIV persistence in the central nervous system. However, in vivo data is crucial to confirm that Cav-1 plays an essential role in establishing HIV persistent infections.

The most widely recognized health problems are vascular complications, including coronary heart disease, pulmonary hypertension, and atherosclerosis [279,280,281,282,283,284,285,286,287,288]. Vascular complications also appear to accelerate in HIV infected patients under ART compared to uninfected cardiac patients [294,295]. A similar cardiac disease risk has also been observed in HIV infected individuals without ART treatment [284,286,288,296]. The influence of HIV on Cav-1 mediated cholesterol efflux in both macrophages and aortic endothelial cells is intriguing and requires further investigation. This may be linked to macrophage foam cell formation and endothelial cell dysfunction with the relatively high incidence of cardiovascular disease seen in HIV infected patients. In addition, the inverse relation of Cav-1 with PAH and the link between PAH and Nef are important areas that need more exploration. Furthermore, the induction of the redistribution of Cav-1 by HIV in endothelial cells requires additional studies to determine the possible consequence on endothelial cell functions including caveolae related mechanosensing. Investigations on whether HIV induced Cav-1 redistribution is due to internalization and/or stretching of caveolae, would enhance such notions and open new venues for understanding HIV pathogenesis.

The higher incidence of risks from Non-AIDS events in HIV infected individuals, especially as they get older, can be related in part to alterations in ROS and antioxidant levels that are noted in numerous tissues in HIV infected individuals [192]. In addition, multiple studies suggest that antiretroviral drugs induce ROS production in HIV infected individuals [192]. Since increased ROS production enhances Cav-1 expression, which is a key player of cellular senescence, there may be a link to non-AIDS related risks with Cav-1 mediated cellular senescence. Thus, up-regulation of the multifunctional Cav-1 by elevated ROS production as a consequence of HIV infection and continuous anti-HIV drug treatments may result in premature cellular senescence accumulation overtime, subsequently causing cellular dysfunction and pathology.

## Figures and Tables

**Table 1:** Caveolin 1/Caveolae functions *.

Functions	Activities
Mechanosensing	Protection of cells from mechanical stress damage and other distress signals.
Lipid Homeostasis	Regulation of membrane lipid composition, cholesterol transport, and efflux.
Endocytosis	Proposed endocytosed cargos include glycosylphosphatidyl inositol (GPI)-anchored proteins, the insulin receptor, shiga and cholera toxins, cholesterol, albumin, and simian virus 40 (SV40). The specificity of caveolae and mechanisms for these cargos are still in question.
Transcytosis	Caveolae transport proteins and lipids such as low density lipoproteins (LDLs) albumin, and insulin from the blood across the interior of the endothelial cells to the opposite face and vice versa.
Signal transduction	Signaling platforms to diverse signaling molecules such as the insulin receptor, endothelial nitric oxide synthase( eNOS,) the epidermal growth factor receptor, and mitogen-activated protein kinase.
Inflammation	Cav-1 modulation of eNOS regulates inflammatory signaling through local control of NO production.
	Ca^2+^ regulation and signaling can also be involved in inflammatory responses.
	Cav-1 serves as a protective protein against cellular stress and inflammation.
Cancer	Serves as a tumor suppresser and at times as an oncogenic factor. Loss of Cav-1 in the stroma is linked to the clinical outcome of breast cancer.
Apoptosis	Involved in supporting multiple anti-apoptotic and survival pathways.
Angiogenesis	Involved in cancer angiogenesis. The Caveolin 1 pathway is also involved in the regulation of vascular endothelial growth factor (VEGF) in association with induced angiogenesis in brain ischemic.
Cellular senescence	Caveolin 1 plays a major role in controlling cellular senescence through its up-regulation by oxidative stress.
Cell cycle regulation	Caveolin 1 is important in the regulation of cyclin D1.

* These functions are reviewed in detail in [94,95,104,111,112,113,114,115,116,117,118,119,120,121,122,123,124,125,127,128,129,130,131,132].
